# Late presentation of Coats disease in a 12-year-old boy: a case report

**DOI:** 10.11604/pamj.2024.49.36.45134

**Published:** 2024-10-11

**Authors:** Ruth Anastasia, Ima Yustiarini, Ady Dwi Prakosa, Sauli Ari Widjaja, Muhammad Firmansjah, Wimbo Sasono

**Affiliations:** 1 Department of Ophthalmology, Faculty of Medicine Universitas Airlangga, Dr Soetomo Hospital, Surabaya, Indonesia

**Keywords:** Coats disease, exudative retinal detachment, retinal telangiectasia, case report

## Abstract

Coats disease is a rare abnormality characterized with retinal telangiectasia and aneurysms with retinal exudation, most often seen in young males and usually affecting only one eye. A 12-year-old boy came in with a three-month history of vision loss and pain in his right eye, alongside progressively worsening blurred vision over the last year. His visual acuity was reduced to only light perception in the right eye, while his left eye maintained 5/5 vision. The intraocular pressure was 43.4 mmHg in the right eye and 15 mmHg in the left eye. Conjunctival hyperaemia, mild corneal edema, iris neovascularization, xantocoria, and dilated pupil was found in anterior segment. Ophthalmoscopy showed retinal telangiectasia in all quadrants and total bullous exudative retinal detachment. Coats disease cases that present at an advanced stage have fewer treatment options and generally a worse prognosis.

## Introduction

Coats disease is defined as unilateral abnormality and exudation of retinal vascular, typically found in young boys. Diagnosing Coats disease remains challenging, as the term has often been misused as a general label for various exudative retinopathies with similar fundus changes [1,2]. The exact pathophysiology of Coats disease remains unknown. Various exudative retinopathies that may resemble this condition include retinitis pigmentosa, retinal macroaneurysms, morning glory anomaly, retinal tumor, ocular toxoplasmosis, and familial exudative retinopathy [1,3]. Therefore, it is crucial to distinguish other exudative retinopathies from Coats disease to alleviate existing confusion surrounding the condition. This case report discusses an advanced case of Coats disease in 12-year-old boy, focusing on the course of the disease.

## Patient and observation

**Patient information:** a 12-year-old boy suffered from vision loss and pain in his right eye over the past three months. He had experienced progressively worsening blurred vision for a year but did not seek medical attention. Within 2 months, throbbing pain was felt in the eyes and became worse. There was no family history of similar conditions. His birth history and growth development were normal. The mother did not undergo Torch screening during pregnancy. The patient denied any history of medication use, trauma, or prior use of glasses. Both his medical history and family medical history were unremarkable.

**Figure 1:**
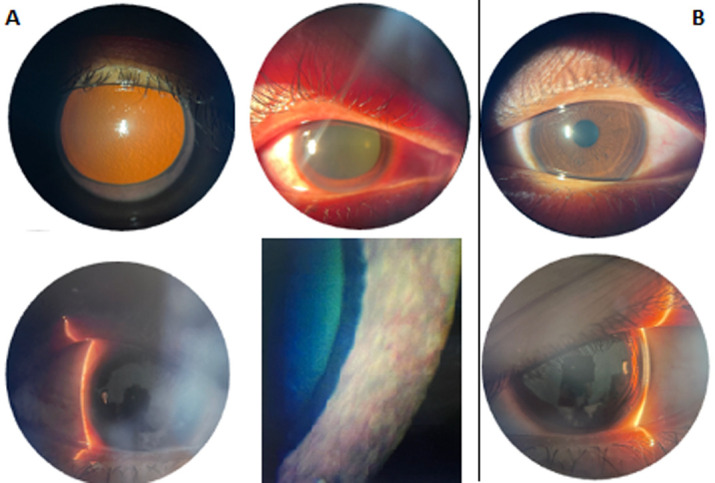
clinical finding of: A) the right eye: conjunctival hyperaemia, slightly oedema cornea, shallow anterior chamber, iris neovascularisation, dilated pupil and xantocoria; B) the left eye: normal finding

**Clinical findings:** upon examination, visual acuity was positive for light perception and 5/5 in the right and left eye, respectively. Intraocular pressure was 43.4 mmHg and 15 mmHg in the right and left eye, respectively. Anterior segment examination of the right eye (Figure 1) revealed conjunctival redness, slight corneal edema, a shallow anterior chamber, iris neovascularization, xantocoria, and a dilated pupil.

**Figure 2:**
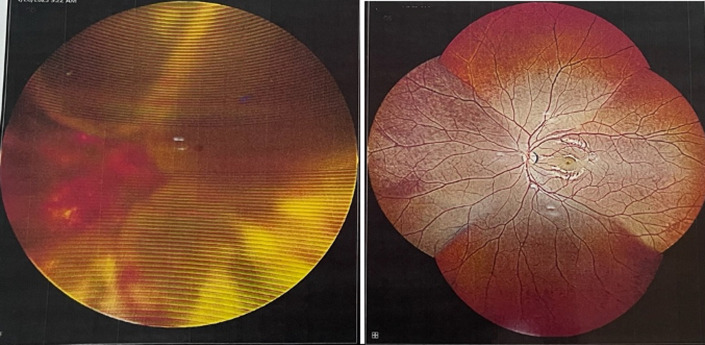
wide field fundus photograph of: A) right eye: bullous exudative total retinal detachment with telangiectasia in all quadrants; B) left eye: normal fundus

**Figure 3:**
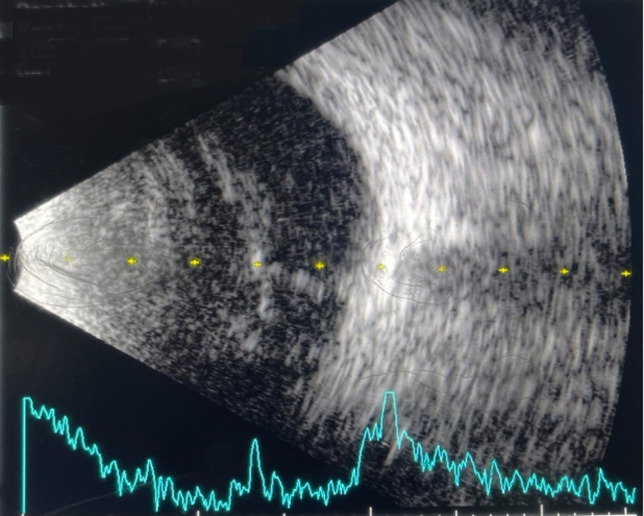
ultrasonography or the right eye revealed membrane-shaped echogenic lesion with minimal mobility and high echo spike with numerous particles beneath the membrane

**Diagnosis assessment:** a wide-field fundus photograph (Figure 2) revealed retinal telangiectasia in all quadrants and total exudative retinal detachment while the left eye appeared normal. Ultrasonography of the right eye identified a membrane-shaped echogenic lesion with minimal mobility and a high echo spike, along with numerous particles beneath the membrane (Figure 3). The patient was then referred to the glaucoma division for management of elevated intraocular pressure. A diagnosis of neovascular glaucoma was then made.

**Diagnosis:** stage IV Coats disease on the right eye.

**Therapeutic interventions:** treatment was initiated to control intraocular pressure with oral acetazolamide 250 mg three times daily, timolol eye drops, and prednisolone eye drops. External drainage and intracameral injection of anti (VEGF) were planned as the surgical management. Unfortunately, the visual acuity had worsened to no light perception a week after and the diagnosis turned into stage V Coats disease. The surgical treatment plan could not be executed and conservative treatment was preferred along with maintaining the intraocular pressure to control the pain and preserve the eyeball.

**Follow-up and outcome of interventions:** intraocular pressure is controlled with the administration of antiglaucoma agents. Pain is reported to be reduced, and regular evaluations of complaints and intraocular pressure are conducted. Evisceration, enucleation, or cyclodestruction may be considered if ocular pain is unmanageable and there are side effects from antiglaucoma medications. For the time being, invasive procedures are not performed considering the young age of the patient and the controlled pain with medication. Regular observation of the fellow eye is also crucial for early detection of abnormalities.

**Patient perspective:** “I have been experiencing progressively blurred vision and occasional pain, but I did not seek medical attention because I felt I could still carry out my activities. However, the worsening pain eventually prompted me to seek treatment. After receiving treatment, the pain has significantly reduced, and I can resume my activities well. My right eye can no longer see, but from this experience, I have learned not to ignore eye complaints and will continue to have regular eye check-ups.”

**Informed consent:** the patient and their family were informed about the case, and they provided both verbal and written consent for the case to be published for the benefit of medical knowledge and public health.

## Discussion

According to established epidemiology and the details of this case, Coats disease is typically diagnosed during the first or second decade of life and predominantly affects young males. In adults, the symptoms of Coats disease can resemble those in children, but they are often less severe. Patients with coats disease found in older age likely had an asymptomatic state during childhood, which was only detected later in life [1,2]. The patient in this case, a 12-year-old boy, experienced chronic vision decline that eventually led to vision loss in one eye. This aligns with the typical age range and disease progression seen in Coats disease patients. Most found cases of Coats disease are unilateral [2]. Bilateral coat disease is uncommon, with a prevalence of less than 10%. The condition exhibits a consistent male predominance and typically affects only one eye, as widely reported in the literature, although no specific explanation has been identified for these patterns [3,4]. Coats disease usually is asymptomatic during the early stages, 60-70% of findings and symptoms occur within the first decade of life. Patients can present with various findings including poor visual acuity, strabismus, xanthocoria, glaucoma, or pain [4,5]. The anterior chamber is commonly found normal. In the late stage, congestive conjunctiva, hazy cornea, rubeosis iridis, and cataracts could be found [1,4]. Visual acuity in Coats disease correlates with the severity and stage of the disease. Visual acuity in these patients can range from 5/5 to complete loss of light perception, with most patients experiencing significant vision loss. In a study by Shields *et al*. visual acuity was documented in 351 cases at presentation, with results showing 22% of patients having 20/40 vision, 30% having 20/50 to 20/200 vision, and 49% with vision less than 20/200 [1,6].

The gold standard for diagnosing Coats disease is a retinal examination using indirect ophthalmoscopy, which reveals retinal telangiectasia in all cases [1,4]. In some patients, multiple zones or quadrants may be involved, displaying a diffuse distribution as in this patient which telangiectasia was found in all quadrants. Ultrasonography of the eye could also be used as a modality to distinguish coats disease from retinoblastoma [1]. Extensive intraretinal exudation and subtotal or total exudative retinal detachment are commonly found in advanced cases as seen in the patient. This patient was presented with total retinal detachment and neovascular glaucoma. Most complications arise as a result of chronic retinal detachment. Patients can develop neovascular glaucoma, characterized by neovascularization of the iris and angle. These patients may experience a painful blind eye, often necessitating enucleation [4,6]. Vitreoretinal surgery can be considered if there is a reasonable chance of achieving retinal reattachment. Concurrent laser photocoagulation or cryotherapy after retinal reattachment helps create a chorioretinal scar [7,8]. There is growing evidence that VEGF levels are elevated in the vitreous and subretinal fluid of eyes affected by Coats disease therefore Anti-VEGF therapy is effective in promoting the regression of abnormal vessels [1,3].

For a painful blind eye, enucleation should be put into consideration. In cases where the eye is blind but not painful, simple observation is recommended [7,9]. Factors that indicate a poor visual prognosis include diffuse zonal involvement, persistent exudation after treatment, retinal macrocysts, and non-Caucasian ethnicity. Given that this patient has diffuse zonal involvement (total exudative retinal detachment) and is of non-Caucasian ethnicity, the prognosis is likely poor [8,10]. Eyes with stage 4 Coats disease or higher are more likely to require enucleation compared to milder stages. There is no significant difference in disease recurrence across stages. In terms of outcomes, more advanced disease stages exhibit lower rates of resolution for the disease, subretinal fluid, and exudation [5,10]. For stage IV Coats disease that has progressed to stage V, as seen in this patient, medical therapy is recommended to manage intraocular pressure and pain in order to preserve the eyeball.

## Conclusion

Coats disease is a retinal abnormality which, although its prevalence is rare, is often found at an advanced stage. Improvements in retinal imaging, diagnostic accuracy, surgical methods, laser therapies, and medical treatments have all enhanced the effectiveness of managing the condition. However, when Coats disease presents at an advanced stage, treatment options become limited, and the prognosis is generally poorer.
